# A novel missense variant of PSEN2 in a Japanese woman with hereditary Alzheimer's disease

**DOI:** 10.1002/alz70860_101422

**Published:** 2025-12-23

**Authors:** Katsuya Nakamura, Kazuki Kasuga, Chinami Yuzawa, Yusaku Shimizu, Norikazu Hara, Takeshi Ikeuchi, Yoshiki Sekijima

**Affiliations:** ^1^ Shinshu University, Matsumoto, Nagano, Japan; ^2^ Ina Central Hospital, Ina, Nagano, Japan; ^3^ Brain Research Institute, Niigata University, Niigata, Niigata, Japan; ^4^ Brain Research Institute, Niigata University, Niigata, Japan

## Abstract

**Background:**

Heterozygous variants of *APP*, *PSEN1*, and *PSEN2* have been reported as causative for hereditary Alzheimer's disease (AD); however, patients with *PSEN2* variants are relatively rare.

**Method:**

We performed clinical and genetic laboratory evaluation of a patient with hereditary AD.

**Result:**

The patient, a 51‐year‐old Japanese woman, was born to non‐consanguineous parents. She had a family history of young onset dementia in her mother. At age 45, her husband first noticed her amnesia, and her cognitive function gradually declined. At age 48, she was referred to our hospital. Results of neuropsychological and neuroradiological studies, including brain MRI and amyloid PET, were consistent with those of AD. By multi‐gene sequencing related to AD, we identified novel heterozygous missense variants in *PSEN2* (c.356T>G, p.Leu119Arg). At age 50, intravenous treatment with lecanemab was initiated.

**Conclusion:**

In this study, we report the first hereditary AD patient with a *PSEN2* variant from Japan, and will discuss the clinical and genetic characteristics of patients with novel PSNE2 variants.

